# Non-contiguous finished genome sequence and description of *Paenibacillus senegalensis* sp. nov.

**DOI:** 10.4056/sigs.3056450

**Published:** 2012-09-24

**Authors:** Ajay Kumar Mishra, Jean-Christophe Lagier, Romain Rivet, Didier Raoult, Pierre-Edouard Fournier

**Affiliations:** Aix-Marseille Université, URMITE, UM63, CNRS7278, IRD198, Inserm1095, Institut Hospitalo-Universitaire Méditerranée Infection, Faculté de médecine,

**Keywords:** *Paenibacillus senegalensis*, genome

## Abstract

*Paenibacillus senegalensis* strain JC66^T^, is the type strain of *Paenibacillus senegalensis* sp. nov., a new species within the genus *Paenibacillus*. This strain, whose genome is described here, was isolated from the fecal flora of a healthy patient. *P. senegalensis* strain JC66^T^ is a facultative Gram-negative anaerobic rod-shaped bacterium. Here we describe the features of this organism, together with the complete genome sequence and annotation. The 5,581,254 bp long genome (1 chromosome but no plasmid) exhibits a G+C content of 48.2% and contains 5,008 protein-coding and 51 RNA genes, including 9 rRNA genes.

## Introduction

*Paenibacillus senegalensis* strain JC66^T^ (= CSUR P157 = DSM 25958) is the type strain of *P. senegalensis* sp. nov. This bacterium was isolated from the stool of a healthy Senegalese patient as part of a “culturomics” study aiming at cultivating all species within human feces, individually. It is a Gram-negative, facultative anaerobic, indole-negative rod.

We recently proposed to include genomic data among other criteria to describe new bacterial species, rather than relying on the poorly reproducible DNA-DNA hybridization and G+C content determination [1]. This strategy creates a polyphasic approach by combining [2] the use of 16S rRNA sequence cutoff values [3] with the plethora of new information provided by high throughput genome sequencing and mass spectrometric analyses of bacteria [4].

Here we present a summary classification and a set of features for *P. senegalensis* sp. nov. strain JC66^T^ together with the description of the complete genomic sequencing and annotation. These characteristics support the creation of the *P. senegalensis* species.

To date, the genus *Paenibacillus* (Ash *et al*. 1994) includes Gram-variable, facultative anaerobic, endospore-forming bacteria, originally classified within the genus *Bacillus* and then reclassified as a separate genus in 1993 [5]. The genus consists of 134 described species and 4 subspecies that have been isolated from a variety of environments including soil, water, rhizosphere, vegetable matter, forage and insect larvae, as well as human specimens [6-9]. The bacteria belonging to this genus produce various extracellular enzymes such as polysaccharide-degrading enzymes and proteases, and have gained importance in agriculture, horticulture, industrial and medical applications [10]. Various *Paenibacillus* spp. also produce antimicrobial substances that are active on a wide spectrum of microorganisms such as fungi, soil bacteria, plant pathogenic bacteria and even important anaerobic pathogens such as *Clostridium botulinum* [11]. In addition, several *Paenibacillus* bacteria can form complex patterns on semi-solid surfaces that require self-organization and cooperative behavior of individual cells by employing sophisticated chemical communication [12]. Pattern formation and self-organization of bacteria within this genus reflect their social behavior and might provide insights into the evolutionary development of the collective action of cells in higher organisms [13]. To the best of our knowledge, this is the first report of isolation of *Paenibacillus sp.* from the normal fecal flora.

## Classification and features

A stool sample was collected from a healthy 16-year-old male Senegalese volunteer patient living in Dielmo (a rural village in the Guinean-Sudanian zone in Senegal), who was included in a research protocol. Written assent was obtained from this individual; no written consent was needed from his guardians for this study because he was older than 15 years old (in accordance with the previous project approved by the Ministry of Health of Senegal and the assembled village population and as published elsewhere [14]. Both this study and the assent procedure were approved by the National Ethics Committee of Senegal (CNERS) and the Ethics Committee of the Institut Fédératif de Recherche IFR48, Faculty of Medicine, Marseille, France (agreement numbers 09-022 and 11-017). The fecal specimen was preserved at -80°C after collection and sent to Marseille. Strain JC66^T^ (Table 1) was isolated in February 2011 after inoculation in Schaedler medium added with kanamycin and vancomycin (BioMerieux, Marcy l’Etoile, France), and incubation at 37°C in aerobic atmosphere.

**Table 1:** Classification and general features of *Paenibacillus senegalensis* strain JC66^T^

**MIGS ID**	**Property**	**Term**	**Evidence code^a^**
	Current classification	Domain *Bacteria*	TAS [15]
		Phylum *Firmicutes*	TAS [16]
		Class *Bacilli*	TAS [17]
		Order *Bacillales*	TAS [18]
		Family *Paenibacillaceae*	TAS [17]
		Genus *Paenibacillus*	TAS [5]
		Species *Paenibacillus senegalensis*	IDA
		Type strain JC66^T^	IDA
	Gram stain	negative	IDA
	Cell shape	rod-shaped	IDA
	Motility	motile	IDA
	Sporulation	sporulating	IDA
	Temperature range	mesophile	IDA
	Optimum temperature	37°C	IDA
MIGS-6.3	Salinity	growth in BHI medium + 5% NaCl	IDA
MIGS-22	Oxygen requirement	facultative anaerobic	IDA
	Carbon source	unknown	
	Energy source	unknown	
MIGS-6	Habitat	human gut	IDA
MIGS-15	Biotic relationship	free living	IDA
MIGS-14	Pathogenicity	unknown	
	Biosafety level	2	
	Isolation	human feces	
MIGS-4	Geographic location	Senegal	IDA
MIGS-5	Sample collection time	September 2010	IDA
MIGS-4.1	Latitude	13.7167	IDA
MIGS-4.1	Longitude	- 16.4167	IDA
MIGS-4.4	Altitude	51 m above sea level	IDA

**^a^**Evidence codes - IDA: Inferred from Direct Assay; TAS: Traceable Author Statement (i.e., a direct report exists in the literature); NAS: Non-traceable Author Statement (i.e., not directly observed for the living, isolated sample, but based on a generally accepted property for the species, or anecdotal evidence). These evidence codes are from the Gene Ontology project [19]. If the evidence is IDA, then the property was directly observed for a live isolate by one of the authors or an expert mentioned in the acknowledgements.

**Figure 1:**
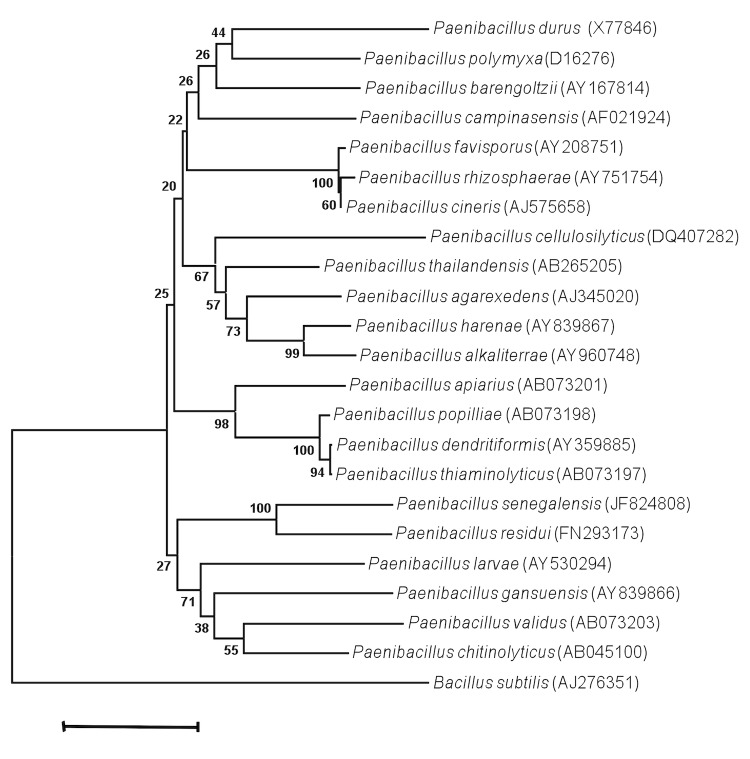
Phylogenetic tree highlighting the position of *Paenibacillus senegalensis* strain JC66^T^ relative to other type strains within the *Paenibacillus* genus. GenBank accession numbers are indicated in parentheses. Sequences were aligned using CLUSTALW, and phylogenetic inferences obtained using the maximum-likelihood method within the MEGA software. Numbers at the nodes are bootstrap values obtained by repeating 500 times the analysis to generate a majority consensus tree. *Paenibacillus subtilis* was used as outgroup. The scale bar represents a 2% nucleotide sequence divergence.

Strain JC66^T^ exhibited a 95.54% nucleotide sequence similarity with *P. residui* [20], the phylogenetically-closest validated *Paenibacillus* species (Figure 1). Although sequence similarity of the 16S operon is not uniform across taxa, this value was lower than the 98.7% 16S rRNA gene sequence threshold recommended by Stackebrandt and Ebers to delineate a new species without carrying out DNA-DNA hybridization [3].

**Figure 2:**
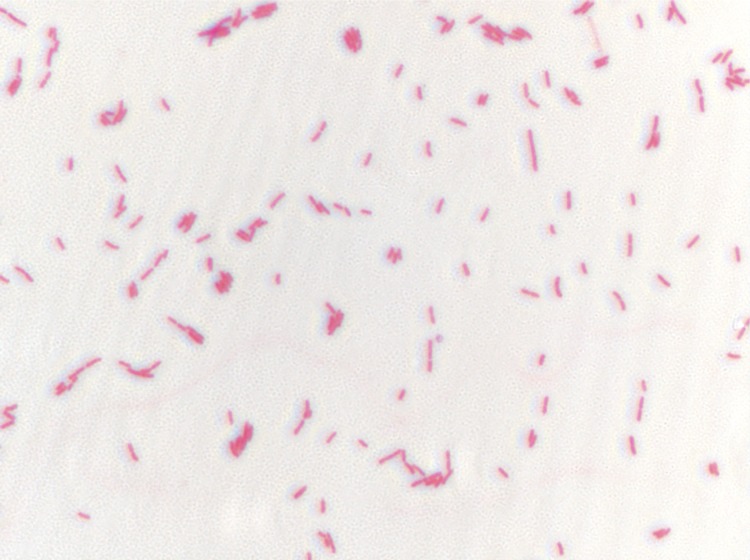
Gram-staining of *P. senegalensis* strain JC66^T^

**Figure 3:**
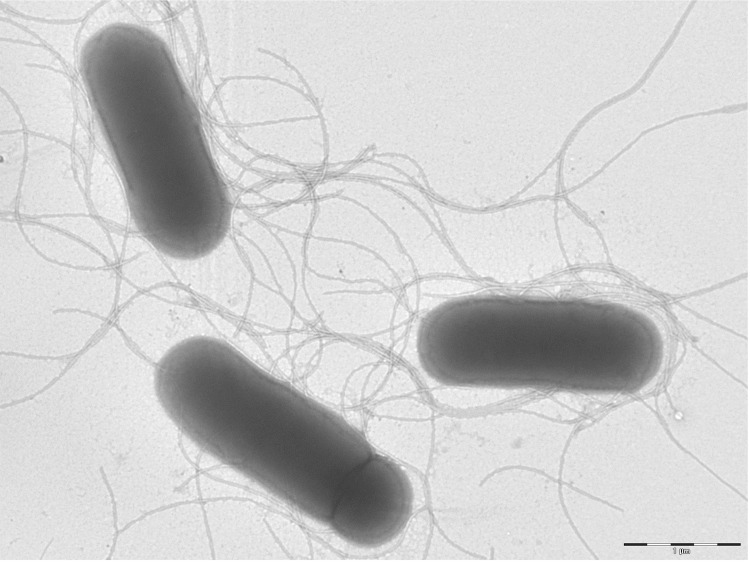
Transmission electron microscopy of *P. senegalensis* strain JC66^T^, using a Morgani 268D (Philips) at an operating voltage of 60kV. The scale bar represents 1 µm.

Growth at different temperatures (25, 30, 37, 45°C) was tested; no growth occurred at 25°C, growth occurred at 30° and 45°C, and optimal growth was observed at 37°C. Translucent and flat colonies were 2 mm in diameter on blood-enriched Columbia agar. Growth of the strain was tested under anaerobic and microaerophilic conditions using GENbag anaer and GENbag microaer systems, respectively (BioMérieux), and in the presence of air, with or without 5% CO_2_. Growth was achieved in aerobic condition with or without CO_2,_ and weak growth was observed in microaerophilic and anaerobic conditions. Gram-staining showed a rod-shaped Gram-negative bacterium (Figure 2). The motility test was positive. Cells showed a mean diameter of 0.66 µm using electron microscopy and exhibited peritrichous flagellae (Figure 3).

Strain JC66^T^ exhibited catalase activity but was negative for indole production. Using API 50CH, positive reactions were observed for D-galactose, D-glucose, D-fructose, D-mannose, and D-sorbitol fermentation. Positive reactions were also observed for N-acteylglucosamine arbutine, esculine, salicine, D-maltose, D-lactose, D-saccharose, D-trehalose, inuline and D-tagatose. Using API ZYM, positive reactions were observed for leucine arylamidase and weak reactions were observed for alkaline phosphatase, esterase lipase, acid phosphatase and naphtol-AS-BI-phosphohydrolase. Using API Coryne, positive reactions were observed for β-glucuronidase, phosphatase alkaline, α-glucosidase, α-galactosidase, and N-acetyl-β-glucosaminidase activities. *P. senegalensis* is susceptible to amoxicillin, ceftriaxone, imipenem, trimethoprim/sulfamethoxazole, ciprofloxacin, rifampin and vancomycin, but resistant to metronidazole.

**Figure 4:**
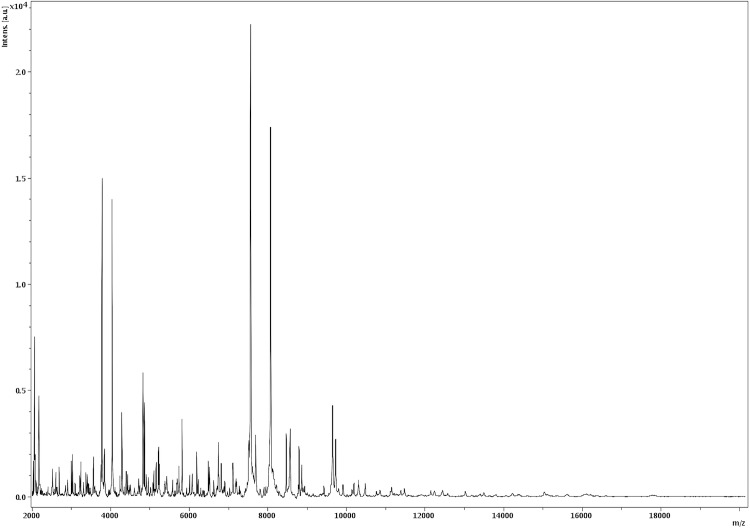
Reference mass spectrum from *P. senegalensis* strain JC66^T^. Spectra from 12 individual colonies were compared and a reference spectrum was generated.

Matrix-assisted laser-desorption/ionization time-of-flight (MALDI-TOF) MS protein analysis was carried out as previously described [21]. Briefly, a pipette tip was used to pick one isolated bacterial colony from a culture agar plate, and to spread it as a thin film on a MTP 384 MALDI-TOF target plate (Bruker Daltonics, Germany). Twelve distinct deposits were done for strain JC66^T^ from twelve isolated colonies. Each smear was overlaid with 2µL of matrix solution (saturated solution of alpha-cyano-4-hydroxycinnamic acid) in 50% acetonitrile, 2.5% tri-fluoracetic acid, and allowed to dry for five minutes. Measurements were performed with a Microflex spectrometer (Bruker). Spectra were recorded in the positive linear mode for the mass range of 2,000 to 20,000 Da (parameter settings: ion source 1 (ISI), 20kV; IS2, 18.5 kV; lens, 7 kV). A spectrum was obtained after 675 shots at a variable laser power. The time of acquisition was between 30 seconds and 1 minute per spot. The twelve JC66^T^ spectra were imported into the MALDI Bio Typer software (version 2.0, Bruker) and analyzed by standard pattern matching (with default parameter settings) against the main spectra of 3,769 bacteria, including spectra from 121 validated *Paenibacillus* species used as reference data, in the Bio Typer database. The method of identification includes the m/z from 3,000 to 15,000 Da. For every spectrum, 100 peaks at most were taken into account and compared with the spectra in database. A score enabled the identification, or not, from the tested species: a score ≥ 2 with a validated species enabled the identification at the species level; a score ≥ 1.7 but < 2 enabled the identification at the genus level; and a score < 1.7 did not enable any identification. For strain JC66^T^, the obtained score was 1.236, thus suggesting that our isolate was not a member of a known species. We incremented our database with the spectrum from strain JC66^T^ (Figure 4).

## Genome sequencing information

### Genome project history

The organism was selected for sequencing on the basis of its phylogenetic position and 16S rRNA similarity to other members of the genus *Paenibacillus*, and is part of a “culturomics” study of the human digestive flora aiming at isolating all bacterial species occurring in human feces. It is the 14^th^ genome of a *Paenibacillus* species and the first genome of *Paenibacillus senegalensis* sp. nov. The Genbank accession number is CAES00000000. Table 2 shows the project information and its association with MIGS version 2.0 compliance.

**Table 2:** Project information

**MIGS ID**	**Property**	**Term**
MIGS-31	Finishing quality	High-quality draft
MIGS-28	Libraries used	One 454 paired end 3-kb library
MIGS-29	Sequencing platforms	454 GS FLX Titanium
MIGS-31.2	Fold coverage	21×
MIGS-30	Assemblers	Newbler version 2.5.3
MIGS-32	Gene calling method	Prodigal
	INSDC ID	PRJEB69
	Genbank ID	CAES00000000
	Genbank Date of Release	January 3, 2012
	Gold ID	Gi13532
MIGS-13	Project relevance	Study of the human gut microbiome

### Growth conditions and DNA isolation

*P. senegalensis* sp. nov. strain JC66^T^, (= CSUR P157 = DSM 25958), was grown on blood agar medium at 37°C. Ten petri dishes were spread and resuspended in 5x100µl of G2 buffer (EZ1 DNA Tissue kit, Qiagen). A first mechanical lysis was performed by glass powder on the Fastprep-24 device (Sample Preparation system) from MP Biomedicals, USA for 40 seconds. DNA was then incubated for a lysozyme treatment (30 minutes at 37°C) and extracted using the BioRobot EZ 1 Advanced XL (Qiagen). The DNA was then concentrated and purified on a Qiamp kit (Qiagen). The yield and the concentration was measured by the Quant-it Picogreen kit (Invitrogen) on the Genios_Tecan fluorometer at 40.2 ng/µl.

### Genome sequencing and assembly

This project was loaded twice on a ¼ region of PTP Picotiterplates for the paired-end sequencing, and once on a 1/8 region for the shotgun sequencing. The shotgun library was constructed with 500 ng of DNA with the GS Rapid library Prep kit (Roche). 5 µg of DNA was mechanically fragmented on the Hydroshear device (Digilab, Holliston, MA, USA) with an enrichment size at 3-4kb. The DNA fragmentation was visualized through the Agilent 2100 BioAnalyzer on a DNA labchip 7500 with an optimal size of 3.7 kb. The library was constructed according to the 454 Titanium paired-end protocol and manufacturer. Circularization and nebulization were performed and generated a pattern with an optimum at 422 bp. After PCR amplification through 15 cycles followed by double size selection, the single stranded paired end library was then quantified on the Quant-it Ribogreen kit (Invitrogen) on the Genios Tecan fluorometer at 180 pg/µL. The library concentration equivalence was calculated as 7.82E+08 molecules/µL. The library was stored at -20°C until further use.

The shotgun library was clonally amplified with 3cpb in 3 emPCR reactions and the paired end library was amplified with lower cpb (1cpb) in 3 emPCR reactions with the GS Titanium SV emPCR Kit (L6ib-L) v2. The yield of the emPCR was 3.52% for the shotgun and 8.01% for the paired-end according to the quality expected by the range of 5 to 20% from the Roche procedure.

Approximately 340,000 beads for the 1/8 region for the shotgun and 790,000 beads on the 1/4 region for the paired-end were loaded on the GS Titanium PicoTiterPlates PTP kit 70×75 and sequenced with the GS Titanium Sequencing Kit XLR70 (Roche). The runs were performed overnight and then analyzed on the cluster through the gsRunBrowser and Newbler assembler (Roche). A total of 370,625 passed filter wells were obtained and generated 115.35Mb with an average length of 312 bp. These sequences were assembled using Newbler with 90% identity and 40bp as overlap. The final assembly identified 197 large contigs (>1500bp) arranged into 18 scaffolds, for a genome size of 5.58Mb, which corresponds to a 21.01 × coverage.

### Genome annotation

Open Reading Frames (ORFs) were predicted using Prodigal [22] with default parameters but the predicted ORFs were excluded if they were spanning a sequencing GAP region. The predicted bacterial protein sequences were searched against the GenBank database [23] and the Clusters of Orthologous Groups (COG) databases using BLASTP. The tRNAScanSE tool [24] was used to find tRNA genes, whereas ribosomal RNAs were found by using RNAmmer [25] and BLASTn against the NR database. Lipoprotein signal peptides and transmembrane helices were predicted using SignalP [26] and TMHMM [27], respectively. ORFans were identified if their BLASTP *E*-value was lower than 1e-03 for alignment length greater than 80 amino acids. If alignment lengths were smaller than 80 amino acids, we used an *E*-value of 1e-05. Such parameter thresholds have already been used in previous works to define ORFans. To estimate the mean level of nucleotide sequence similarity at the genome level between *Paenibacillus* species, we compared the ORFs only using BLASTN and the following parameters: a query coverage of ≥ 70% and a minimum nucleotide length of 100 bp. Artemis [28] was used for data management and DNA Plotter [29] was used for visualization of genomic features. Mauve alignment tool was used for multiple genomic sequence alignment [30].

## Genome properties

**Figure 5:**
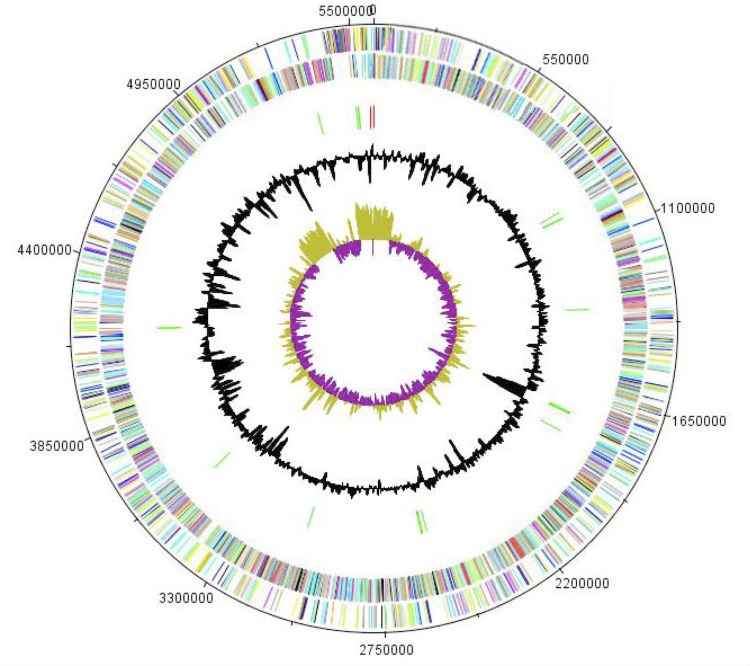
Graphical circular map of the chromosome. From the outside in, the outer two circles shows open reading frames oriented in the forward (colored by COG categories) and reverse (colored by COG categories) direction, respectively. The third circle marks the rRNA gene operon (red) and tRNA genes (green). The fourth circle shows the G+C% content plot. The inner-most circle shows GC skew, purple indicating negative values whereas olive for positive values.

The genome of *P. senegalensis* sp. nov. strain JC66^T^ is 5,581,254 bp long (1 chromosome but no plasmid) with a 48.2% G + C content of (Figure 5 and Table 3). Of the 5,059 predicted genes, 5,008 were protein-coding genes, and 51 were RNAs. Nine rRNA genes (three 16S rRNA, three 23S rRNA and three 5S rRNA) and 42 predicted tRNA genes were identified in the genome. A total of 3,588 genes (71.00%) were assigned a putative function. Five hundred and four genes were identified as ORFans (10%). The remaining genes were annotated as hypothetical proteins. The properties and statistics of the genome are summarized in Table 3. The distribution of genes into COGs functional categories is presented in Table 4.

**Table 3:** Nucleotide content and gene count levels of the genome

Attribute	Value	% of Total^a^
Size (bp)	5,581,254	
G+C content (bp)	2,690,164	48.2
Coding region (bp)	4,693,047	84.08
Number of replicons	1	
Extrachromosomal elements	0	
Total genes	5,059	100
RNA genes	51	0.89
rRNA operons	3	
Protein-coding genes	5,008	99.10
Genes with function prediction	3,805	75.30
Genes assigned to COGs	3,588	71.00
Genes with peptide signals	366	7.30
Genes with transmembrane helices	1,407	27.84

^a^ The total is based on either the size of the genome in base pairs or the total number of protein coding genes in the annotated genome

**Table 4:** Number of genes associated with the 25 general COG functional categories

**Code**	**Value**	**%age**^a^	**Description**
J	181	3.61	Translation
A	0	0	RNA processing and modification
K	458	9.14	Transcription
L	180	3.59	Replication, recombination and repair
B	1	0.02	Chromatin structure and dynamics
D	36	0.72	Cell cycle control, mitosis and meiosis
Y	0	0	Nuclear structure
V	122	2.44	Defense mechanisms
T	231	4.61	Signal transduction mechanisms
M	220	4.39	Cell wall/membrane biogenesis
N	53	1.06	Cell motility
Z	2	0.04	Cytoskeleton
W	0	0	Extracellular structures
U	49	0.98	Intracellular trafficking and secretion
O	102	2.03	Posttranslational modification, protein turnover, chaperones
C	154	3.07	Energy production and conversion
G	507	10.12	Carbohydrate transport and metabolism
E	318	6.35	Amino acid transport and metabolism
F	81	1.61	Nucleotide transport and metabolism
H	133	2.65	Coenzyme transport and metabolism
I	84	1.68	Lipid transport and metabolism
P	239	4.78	Inorganic ion transport and metabolism
Q	81	1.62	Secondary metabolites biosynthesis, transport and catabolism
R	573	11.44	General function prediction only
S	304	6.07	Function unknown
-	1,420	28.35	Not in COGs

^a^ The total is based on the total number of protein coding genes in the annotated genome.

## Comparison with the genomes from other *Paenibacillus* species

**Figure 6:**
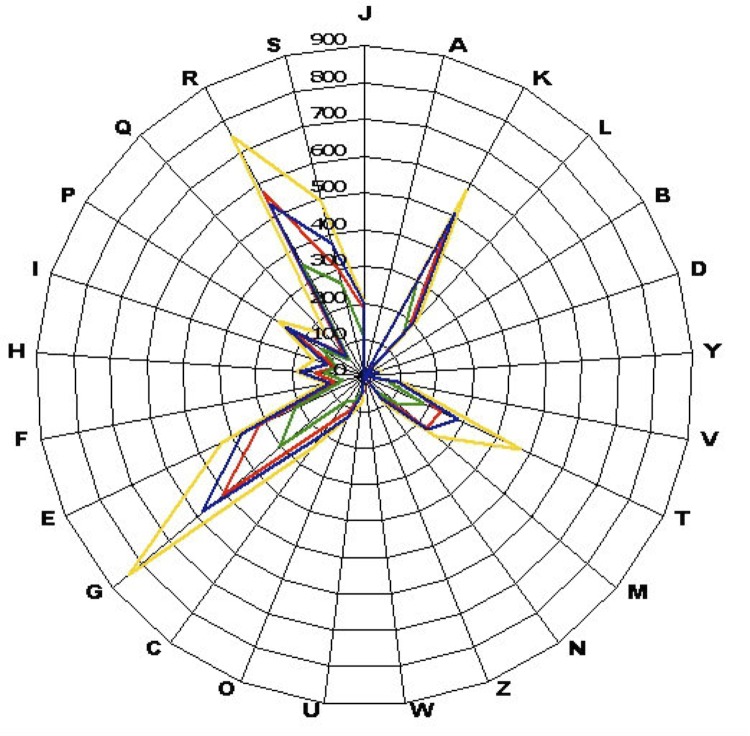
Compared distribution of genes in COGs functional categories in *P. senegalensis* (colored in red), *P. terrae* (colored in blue), *P. mucilaginosus* (colored in yellow) and *P. polymyxa* (colored in green) chromosomes.

To date, the genomes of three validated *Paenibacillus* species are available. Here, we compared the genome sequence of *P. senegalensis* strain JC66^T^ with those of *P. terrae* strain HPL-003 (GenBank accession number NC_016641.1), *P. polymyxa* strain M1 (NC_017542.1) and *P. mucilaginosus* strain 3016 (NC_016935.1). The *P. senegalensis* genome has a similar size to that of *P. polymyxa* (5.58 Mb *vs* 5.73 Mb, respectively) but is smaller than those of *P. terrae* and *P. mucilaginosus* (6.08 and 8.74 Mb, respectively). The G+C content of *P. senegalensis* is higher than those of *P. polymyxa* and *P. terrae* (48.2, 44.8 and 46.8%, respectively) but smaller than *P. mucilaginosus* (58.31%). The gene content of *P. senegalensis* is larger than those of *P. polymyxa* (5,059 and 3,602, respectively) but smaller than those of *P. terrae* and *P. mucilaginosus* (6,414 and 5,642, respectively). The ratio of genes per Mb of *P. senegalensis* is larger than those of *P. mucilaginosus* and *P. polymyxa* (906, 861 and 578, respectively) but smaller than that of *P. terrae* (928). The gene distribution into COG categories is very similar in all four compared genomes (Figure 6).

*P. senegalensis* shares mean degrees of sequence similarity at the genome level of 81.5% (range 70.34-100%), 80.6% (range 70.46-100%) and 81.32% (range 70.34-100%) with *P. polymyxa*, *P. mucilaginosus* and *P. terrae*, respectively.

## Conclusion

On the basis of phenotypic, phylogenetic and genomic analyses, we formally propose the creation of *Paenibacillus senegalensis* sp. nov. that contains the strain JC66^T^. This bacterium has been found in Senegal.

### Description of *Paenibacillus senegalensis* sp. nov.

*Paenibacillus senegalensis* (se.ne.gal.e’n.sis. L. gen. masc. n. *senegalensis*, pertaining to Senegal, the country from which the specimen was obtained).

Colonies are 2 mm in diameter on blood-enriched Columbia agar. Cells are rod-shaped with a mean diameter of 0.66 μm. Optimal growth is achieved in aerobic condition with or without CO_2_. Weak growth is observed in microaerophilic and anaerobic conditions. Growth occurs between 30 and 45°C, with optimal growth observed at 37°C, on blood-enriched agar. Cells are Gram-negative, endospore-forming, and motile. Cells are catalase positive but negative for indole production. D-galactose, D-glucose, D-fructose, D-mannose, D-sorbitol, N-Acetylglucosamine arbutine, esculine, salicine, D-maltose, D-lactose, D-saccharose, D-trehalose, inuline, D-tagatose, β-glucuronidase, phosphatase alkaline, α-glucosidase, α-galactosidase, and N-acetyl-β-glucosaminidase metabolic activities are present. Weak alkaline phosphatase, esterase lipase, acid phosphatase and naphtol-AS-BI-phosphohydrolase activities are observed. Cells are susceptible to amoxicillin, ceftriaxone, imipenem, trimethoprim/sulfamethoxazole, ciprofloxacin, rifampin and vancomycin, but resistant to metronidazole. The G+C content of the genome is 48.2%. The 16S rRNA and genome sequences are deposited in Genbank under accession numbers JF824808 and CAES00000000, respectively. The type strain is JC66^T^ (= CSUR P157 = DSM 25958) was isolated from the fecal flora of a healthy patient in Senegal.
